# Seeing Patterns—from Glass Art to Public Health

**DOI:** 10.3201/eid3208.AC3208

**Published:** 2026-08

**Authors:** Lesli Mitchell, Ermias Belay

**Affiliations:** Centers for Disease Control and Prevention, Atlanta, Georgia, USA

**Keywords:** Ermias Belay, glass art, Mondrian, art-science connection, public health

**Figure:**
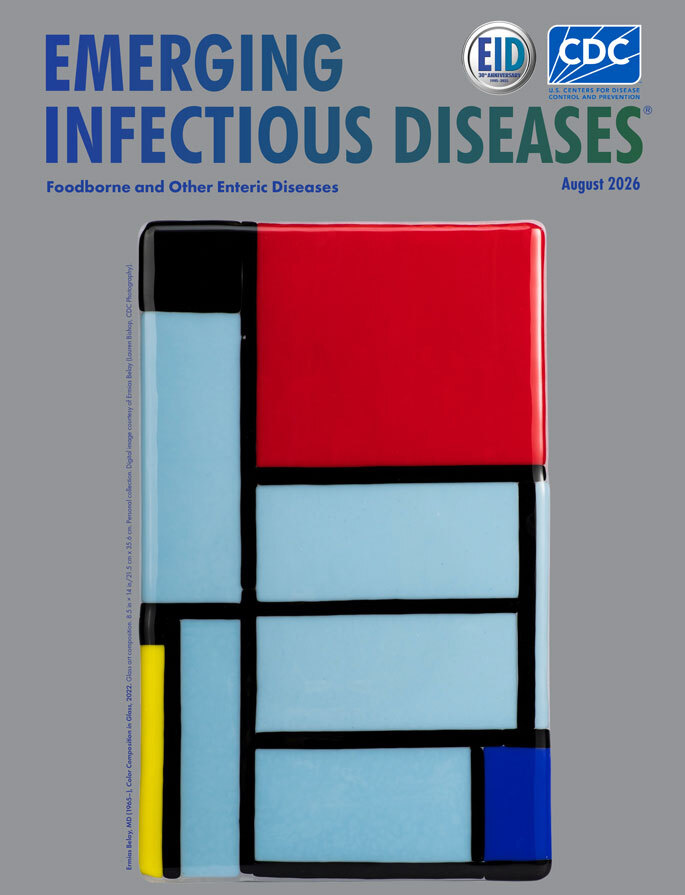
**Ermias Belay, MD (1965–), *Color composition in glass,* 2022.** Glass art composition. 8.5 × 14 in/21.5 cm x 35.6 cm. Personal collection. Digital image courtesy of Ermias Belay (Lauren Bishop, CDC Photography).

I wish to approach truth as closely as is possible, and therefore I abstract everything until I arrive at the fundamental quality of objects.

—Piet Mondrian (*1*)

The cover of this month’s *Emerging Infectious Diseases* features *Color composition in glass* by Dr. Ermias Belay, Acting Director of the Public Health Infrastructure Center at the Centers for Disease Control and Prevention. Glassworking traces its roots to ancient Egypt and Assyria (*2*,*3*). Early glassworking in Egypt began in the 3rd millennium BCE, during the Early Dynastic Period, and used a method known as core-forming. That type of glassworking used a core of clay or sand that was shaped, coated with molten glass, and manipulated while hot to achieve the desired shape. After cooling, the core was removed, leaving behind a hollow glass vessel. 

The first glass objects varied in color and transparency and were used mainly for simple beads, decorative elements, and inlays. In its earliest forms, glass objects or vessels were limited to those in the most affluent social circles. In fact, early glassmaking workshops in Mesopotamia, Egypt, and the Aegean were generally associated with palaces or temples because of the material’s exclusive status. That status continued during the Middle Ages, when colored glass was made by artisans for stained glass windows in Gothic cathedrals (*4*).

By the late Middle Ages, experimentation in glass techniques emphasized artistic effects. During the 13th Century, the island of Murano became known for its skilled glassmakers and artistic glasswork, including clear cristallo glass, gold and colored enamel decoration, milky lattimo glass, opalescent effects, and filigree designs (*5*). Murano glass continues to maintain its popularity, and its centuries-long influence can be seen in specialty glass companies of the 18th and 19th Centuries, such as Tiffany, Lalique, Daum, and Gallé. The mid-20th Century saw the rise of the Studio Glass Movement, thanks to the development of small, inexpensive furnaces that could be used in the studio. That development “changed the glassmaking game” and enabled individual artists and small collectives to design and produce glass art (*6*).

Throughout his life, Belay has appreciated a wide range of art forms, particularly paintings, but he found his talents and creative interest best expressed through glass art. Belay’s personal experience with fine glass art influenced his choice to work in glass:

Glass is just a beautiful material to work with and the finished product is usually exquisite. My fascination with glass increased after visiting Murano, Italy, more than 15 years ago. Observing the artisans in Murano craft exquisite glass objects left a lasting impression on me and deepened my admiration for the material.

Belay was drawn to working with glass because of its unique ability to merge vibrant color with translucence in a way that enables each piece to interact dynamically with light. Glass reflects and refracts light in different ways depending on its color, translucence, and interaction with its surroundings. For Belay, the combination of color, transparency, and the effect of illumination makes creating glass art a fulfilling and inspiring pursuit, and he has been creating art glass pieces for nearly 10 years.

*Color composition in glass* was created in 2022. In total, Belay spent ≈23 hours on the work: 15 hours to create the design, cut the glass pieces (the most challenging part for Belay), and smooth them out; and an additional 8 hours in the kiln to fuse the glass pieces together into the final work. The piece is influenced by the style of Piet Mondrian, one of the founders of the Dutch modern movement, De Stijl, which was both a reaction to Art Deco and response to the aftermath of World War II in that it strove to provide a sense of order and balance. De Stijl embraced an abstract, elemental aesthetic centered in basic visual elements such as geometric forms and primary colors. In his paintings during the 1920s, for which he is best known, Mondrian’s abstract lines and rectangles, as well as his simple palette, represented objects at their most fundamental level. For Mondrian, this level of abstraction represented the “truth” of an object and resonated with the aims of the movement to reflect the spiritual order underlying the physical world.

Belay explains Mondrian’s influence on this piece:

The simplicity of his work by reducing objects into their basic forms of line, color, and graphics is very inspiring. My love of colors in art is very nicely expressed in his many works of composition in red, yellow, and blue. This is one of the pieces I cherish. I deliberately avoided making it into any shape to mimic the work Mondrian did with his rectangular pieces.

Although the work was created for Belay’s personal use, and he says he has not had time to publicly exhibit his glass art, he hopes to mount an exhibit in the future. It’s not surprising that Belay has not had much time for art exhibits. His career at the Centers for Disease Control and Prevention has led to national and international recognition as an expert in prion diseases and global health, and he has made substantial contributions to public health through more than 150 publications in scientific journals with outstanding scientific impact, including numerous contributions to *Emerging Infectious Diseases*.

The qualities that attract Belay to glass art also underpin the scientific work of public health, including careful attention to detail, appreciation of complex patterns, and the ability to reveal structure through color and light. Just as individual pieces of glass are assembled into a coherent composition, food safety depends on understanding the interconnected systems that link food production, processing, distribution, and consumption across the globe. This month’s theme highlights how those connections can create pathways for foodborne hazards to spread across borders and emphasizes continued research and surveillance. We are reminded that the risks for emerging infectious diseases often require recognizing that what is not immediately apparent, whether in art or science, can illuminate hidden patterns and connections. This perspective is particularly important for enteric disease surveillance, where integrating human, animal, and environmental data through a One Health approach can reveal transmission pathways, detect emerging threats earlier, and inform more effective prevention and response strategies. In public health, such careful observations are critical for understanding risks and protecting health.
